# The acquisition of long-lived memory B cell responses to merozoite surface protein-8 in individuals with *Plasmodium vivax* infection

**DOI:** 10.1186/s12936-019-2821-z

**Published:** 2019-05-31

**Authors:** Piyawan Kochayoo, Natthapon Kittisenachai, Siriruk Changrob, Kittikorn Wangriatisak, Fauzi Muh, Patchanee Chootong, Eun-Taek Han

**Affiliations:** 1 Department of Clinical Microbiology and Applied Technology, Faculty of Medical Technology, Mahidol University, Bangkok, 10700 Thailand; 2 Department of Medical Environmental Biology and Tropical Medicine, School of Medicine, Kangwon National University, Chuncheon, Gangwon-do 24341 Republic of Korea

**Keywords:** *Plasmodium vivax*, Merozoite surface protein 8, Memory B cells

## Abstract

**Background:**

The ability of a malaria antigen to induce effective, long-lasting immune responses is important for the development of a protective malaria vaccine. *Plasmodium vivax* merozoite surface protein-8 (PvMSP8) has been shown to be immunogenic in natural *P. vivax* infections and produces both cell-mediated and antibody-mediated immunity. Thus, PvMSP8 has been proposed as a vaccine candidate following fusion with other merozoite antigens in blood stage vaccine design. Here, the long-term responses of antibodies and memory B cells (MBCs) specific to PvMSP8 in individuals were monitored in a longitudinal cohort study.

**Methods:**

Both cross-sectional surveys and cohort studies were utilized to explore the persistence of antibody and MBC responses to PvMSP8. Antibody titers were detected in individuals with acute disease and those who recovered from an infection for 4 years. The dominant peptide epitope of PvMSP8 recognized by naturally acquired antibodies was examined to observe the durability of the post-infection antibody response. PvMSP8-specific MBCs were also in subjects 4 years post-infection using an enzyme-linked immunospot assay.

**Results:**

The prevalence of antibodies to PvMSP8 was high during and after infection. The antibody levels in individual responders were monitored for up to 12 months post-infection and showed that most patients maintained their seropositive response. Interestingly, the anti-PvMSP8 antibody responses stably persisted in some patients who had recovered from an infection for 4 years. Positive PvMSP8-specific MBCs were also detected at 4 years post-infection. However, analysis in these individuals showed no correlation with the presence or titer of circulating antibody.

**Conclusion:**

PvMSP8 had the ability to induce a long-term humoral immune response. The antibodies and MBCs specific for this antigen developed and persisted in subjects who acquired a natural *P. vivax* infection. Inclusion of the PvMSP8 antigen in blood stage vaccine design should be considered.

## Background

Highly effective malaria vaccines are required for use as part of a repertoire of tools for the elimination or eradication of malaria. Malaria vaccines are generally classified based on their target within the parasite lifecycle: (i) a pre-erythrocytic vaccine aims to prevent blood stage infections, (ii) a blood stage vaccine aims to clear parasitaemia and prevent clinical disease, and (iii) a transmission-blocking vaccine aims to prevent the infection of mosquitoes and interrupt the malaria transmission cycle [1]. The leading vaccine candidate against the blood stage of *Plasmodium vivax* is Duffy binding protein region II (DBPII), the only candidate antigen that has currently reached clinical trials [2]. However, an obstacle to this vaccine’s efficacy is strain-specific immunity due to the presence of DBPII allelic variation [3]. The identification of conserved epitopes among DBPII variants has become a substantial challenge in the development of DBPII-based vaccines. Moreover, the increasing recognition of *P. vivax* infections in Duffy-negative individuals suggests that there is an alternative pathway for the parasites to invade red blood cells. Together, this suggests that a combination of antigens would enhance the efficacy of a vivax malaria vaccine and more likely lead to broadly protective immune responses.

Merozoite surface protein 8 (MSP8) is a glycosylphosphatidylinositol (GPI)-anchored protein expressed in blood stage malaria parasites. MSP8 possesses two epidermal growth factor (EGF)-like domains at the C terminus, and these modules are considered potential targets of protective immunity. High antigenicity of MSP8 has been shown in both *Plasmodium yoelii* and *Plasmodium falciparum*. In *P. yoelii*, immunization with full-length MSP8 fused with MSP1-19 induced a T cell response and high levels of protective antibodies against these two antigens [4]. *Plasmodium falciparum* MSP8 (PfMSP8) was found to be immunogenic during natural infections. Sera from malaria-exposed patients appear to recognize B cell epitopes within the first 350 amino acids of PfMSP8 that contain an Asn/Asp-rich domain [5, 6]. A recombinant PfMSP8 protein induced strong T and B cell responses in immunized mice [7]. Importantly, immunization with an MSP8/merozoite surface protein 1 (MSP1) chimera elicits an antibody response that inhibits *P. falciparum* blood stage growth in vitro, indicating that MSP8 represents a potential malaria-specific carrier protein to enhance the immunogenicity of neutralizing B cell epitopes in the 19-kDa C terminal domain of MSP1 [7–9]. Based on the results from limited studies of *P. vivax*, patients predominantly produce the IgG2 antibody subtype against MSP8 protein. The total IgG response to PvMSP8 increased up to day 7 and then decreased slightly within a month. Anti-PvMSP8 antibodies are stably sustained for up to 12 years post-recovery in patient samples from regions of China where malaria is no-longer endemic [10]. Most anti-PvMSP8 antibodies present in positive responders recognize two epitopes located outside of the C terminal EGF-like domain. Furthermore, the cellular immune response in *P. vivax*-infected exposed individuals produces high levels of IFN-γ and IL-10 upon PvMSP8 antigen stimulation in vitro [10]. Together, these reports indicate an ability of this protein to trigger both cellular and humoral immunity. However, further characterization of the ability of PvMSP8 to stimulate long-term immune responses is required before this antigen is proposed as a vaccine candidate in combination with other blood stage antigens in malaria vaccine development.

The memory immune response is an essential component of adaptive immunity to infective agents, including *Plasmodium* spp. The persistence of malaria-specific memory B cells (MBCs) was shown in individuals who lived in regions with low malaria endemicity, indicating that long-lived MBCs were developed and maintained in the absence of re-infection [11, 12]. In contrast, a short-term response has been observed in regions with high malaria endemicity; a response which involved the expansion of atypical MBC subsets resulting in short-lived antibody responses [13, 14]. In this study, the ability of the PvMSP8 antigen to induce long-term humoral immunity in the absence of parasite stimulation was explored using cohort studies. The development and persistence of antibody responses were assessed in individuals during the acute disease and recovery phase of *P. vivax* infections. Long-term responses of PvMSP8-specific MBCs were also observed in *P. vivax*-infected patients who had recovered from an infection acquired 4 years previously. This study contributes to a better understanding of the long-term humoral immune response to *P. vivax* and will be useful in the development of effective malaria vaccines in the future.

## Methods

### Study site and sample collection

Both cross-sectional and longitudinal analyses were designed to evaluate the long-term antibody and memory B cell responses to the PvMSP8 antigen following natural *P. vivax* exposure at Rap Ro, a village near the Myanmar border of Southern Thailand where malaria transmission is seasonal and peaks during the rainy season (May to December). Firstly, the seroprevalence of antibody to PvMSP8 was assessed using a cross-sectional study, between May 2014 and May 2017. Plasma samples were collected from *P. vivax*-infected subjects during the acute (n = 40) and recovery phases of infection [at 3 months (3 months, n = 35), 9 months (9 months, n = 26), and 12 months (12 months, n = 25)] to measure antibody levels using an indirect ELISA (Table 1). Of the 40 acutely infected patients, 16 patients were available for sample collection at all time points. These patients were included in the sub-cohort 1 study designed to assess the longevity of antibody responses to recombinant protein and synthesized peptides of PvMSP8 (Table 2). To further explore the long-term antibody responses against this protein post-infection, 5 subjects from the total 40 infected patients were further monitored up to 12 months post-infection for anti-PvMSP8 antibody as the sub-cohort 2 study (between May 2014 and May 2018). Finally, the persistence of seropositive anti-PvMSP8 responses at 4 years post-infection was investigated by assessing specific MBC responses in 13 other patients who had recovered from a *P. vivax* infection 4 years earlier (Table 4).

**Table 1:** Characteristics of study participants in survey of PvMSP8 antigenicity at different time points after *P. vivax* infection

Characteristics	*P. vivax*-infected participants			
	Acute vivax patients	Recovered subjects		
		3 months	9 months	12 months
Total (n)	40	35	26	25
Gender				
Male	28	24	15	16
Female	12	11	11	9
Age Mean ± SD	37.4 ± 12	40.0 ± 1.4	41.2 ± 12.2	41.1 ± 11.8
Parasitaemia (parasite/µL) Mean ± SD	5170.8 ± 5238.3	0	0	0
No. of prior infections				
0	40	35	25	24
1	0	0	1	1
> 1	0	0	0	0
No. of recorded re-infections	0	0	1	1

**Table 2:** The history of *P. vivax* infections in subjects enrolled in the antibody longevity study followed for 12 months

Total number	No. of positive responders at acute phase (%)	Ages	Gender		No. of prior infections			No. of recorded re-infections	
		Mean ± SD	Male	Female	0	1	> 1	Symptomatic infection	Asymptomatic infection
16	16 (100%)	44.0 ± 9.35	12	4	15	1	0	1	0

Blood samples used as malaria-naïve controls (n = 20) were obtained from healthy volunteers who lived in non-malariaous areas and had no history of exposure to *Plasmodium* parasites. Venous blood samples were collected in Vacuette Heparin tubes (Greiner Bio-One, Monroe, NC, USA) and transported to the laboratory within 6 h. Thick and thin blood smears, as well as nested PCR, were used to determine the presence of *P. vivax* parasites. Historical data of previous malaria infections and the number of *P. vivax* infections in study subjects were obtained from the records of the Vector Borne Disease Unit, based on blood smear and nested PCR detection. Patients were scheduled for follow-up blood collection every 3 months to assess sub-patent malaria by nested PCR. Staff in this unit conducted weekly house-to-house visits from May 2014 to May 2017 (sub-cohort 1) or May 2014 to May 2018 (sub-cohort 2) to estimate the incidence of clinical malaria over the study period. Ethical approval was obtained from the Committee on Human Rights Related to Human Experimentation, Mahidol University, Thailand (MUIR 2012/079.2408).

### Expression of the recombinant PvMSP8 protein

The recombinant PvMSP8 protein was expressed as previously described [10]. Briefly, *P. vivax* DNA was amplified using the specific primers PvMSP8F 5′-GGGCGGATATCTCGAGGGAAACGTTAGCCCACCC-3′; and PvMSP8R 5′-GCGGTACCCGGGATCCTTAGCAGTATATTCCGTCTCCCTCA-3′. Then, this PvMSP8 DNA was cloned into the pEU-E01-His-TEV-MCS vector (CellFree Sciences, Matsuyama, Japan) and expressed using a WGCF expression system. The protein was purified using a Ni–NTA agarose column (Qiagen, Hilden, Germany). The production of rPvMSP8 protein was detected by western blotting using an anti-penta-His antibody (Qiagen).

### Detection of the total IgG response to PvMSP8

Anti-PvMSP8 antibody levels in plasma samples from patients with acute *P. vivax* infections and follow-up samples collected 3 months, 9 months, 12 months, 3 years and 4 years after recovery were assayed using an enzyme-linked immunosorbent assay (ELISA). Briefly, 2 µg/mL rPvMSP8 protein was coated onto 96-well plates and incubated at 4 °C overnight. The plates were then incubated with blocking buffer for 2 h at room temperature (RT) following which 100 μL of plasma diluted 1:200 in blocking buffer were added to each well and incubated for 1 h. After washing the plates five times, a 1:1000 dilution of goat anti-human IgG-alkaline phosphatase in blocking buffer was added to each well. Finally, the 2,2-azino-bis(3-ethylbenzthiazoline-6-sulfonic acid) substrate (Sigma-Aldrich, St. Louis, MI, USA) was added to detect antigen–antibody reactivity. Absorbance was recorded at 405 nm 1 h after the addition of the developer reagent. OD values for duplicate wells for each sample were averaged. A baseline OD was established using plasma samples from 20 Thai individuals who were not exposed to malaria; this control value was subtracted from the test OD values to standardize the assay.

### Analysis of long-term antibody responses induced by the PvMSP8 peptides

Four peptides were recognized by anti-PvMSP8 antibodies in our previous study [10]. These four synthesized peptides (Peptide No. 2: DDSFDLSDYLADFELINY, Peptide No. 3: LADFELINYIIMHETSEL, Peptide No. 4: IIMHETSELIDELINIIE and Peptide No. 5: IDELLNIIESMNFRKESG) were used to detect specific antibodies in study samples in the 3-year and 4-year cohort studies, and to determine whether these specific epitopes of PvMSP8 induced long-term responses following a natural infection. A set of 18-mer peptides spanning the conserved C terminus of PvMSP8 Sal-1 sequences, each overlapping by nine amino acids, were custom synthesized at > 90% purity, purified, and used for the coating step of the ELISA (Peptron Co., Ltd., Daejeon, Korea). Peptides were coated onto 96-well plates at a concentration of 10 μg/mL and incubated at 4 °C overnight. Then, the IgG responses to peptides were detected using the protocol for the detection of anti-rPvMSP8 antibodies. The cut-off value was calculated as the mean plus two SDs of the ODs of the plasma samples from the 20 healthy controls (HC).

### Enzyme-linked immunospot assay (ELISPOT)

Peripheral blood mononuclear cells (PBMCs) were adjusted to a concentration of 1 × 10^6^ cells/mL and distributed into 24-well culture plates. Then, cells were stimulated with a mixture of 1 μg/mL R848 and 10 ng/mL rhIL-2 (Mabtech, Stockholm, Sweden), and cultured with 5% CO_2_ at 37 °C for 3 days. The 96-well ELISPOT plates (Millipore) were coated with 10 μg/mL rPvMSP8 or 1 mg/mL of tetanus toxoid (TT) or 15 μg/mL mAbMT91/145 (anti-human IgG monoclonal antibody) in PBS at 4 °C overnight to detect cells secreting PvMSP8-specific antibodies. The wells were blocked with R10 (10% FBS in RPMI 1640 media) at RT for 1 h. Stimulated PBMCs were harvested, washed and incubated in PvMSP8 antigen-coated ELISPOT wells for 16–24 h. The wells were washed and incubated with 100 μL of 1 μg/mL mAb MT78/145 (biotinylated anti-human IgG monoclonal antibodies) at RT for 2 h. After washes with PBS, 100 μL of streptavidin-conjugated HRP was added and incubated at RT for 1 h. The plate was washed and 100 μL of TMB substrate solution were used to detect the spots. After 10 min, the enzymatic reaction was stopped by rinsing the plate with deionized water. Spots were counted using a Bioreader 5000 Pro-F gamma ELISPOT Reader (BioSys GmbH, Germany). A positive response of antigen-specific MBCs was defined as spot-forming cells (SFCs) with a twofold or greater total number of spots than that of the negative controls.

### Statistical analysis

Total IgG responses to recombinant PvMSP8 or synthesized peptides were compared between unpaired groups (acute disease compared to recovery, or patient compared to healthy controls) using the Mann–Whitney *U* test. Statistical analysis was performed and graphs prepared using GraphPad Prism software (v. 5; GraphPad Software, San Diego, CA, USA).

## Results

### Prevalence of anti-PvMSP8 IgG levels in the acute and recovery phases of *P. vivax* infections

**Fig. 1:**
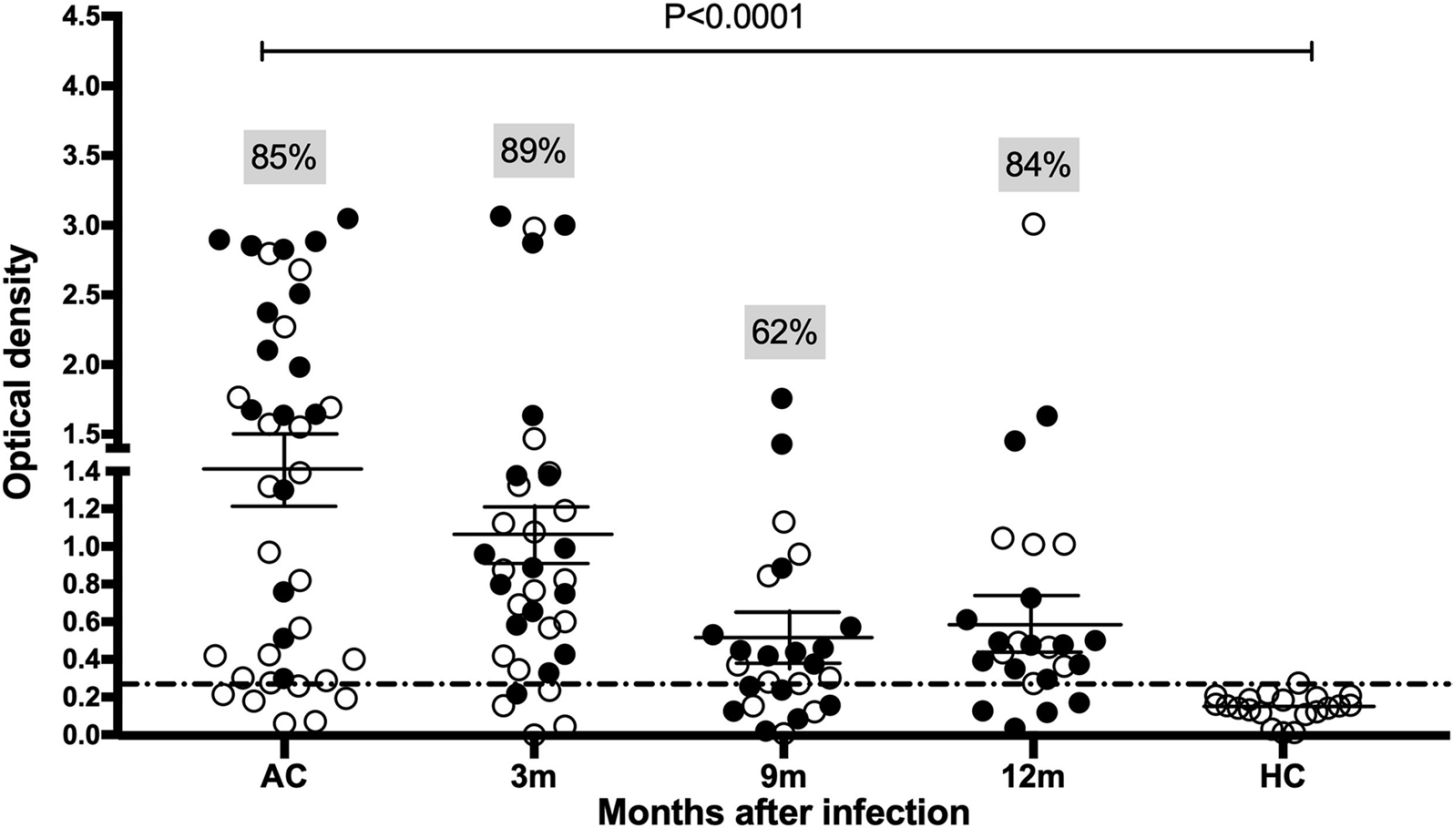
Antibody responses to PvMSP8 in the acute disease stage and after infection. A cross-sectional survey was used to explore the seroprevalence of the antibody response in symptomatic patients with a *P. vivax* infection (n = 40) and after recovery from infection for 3 (n = 35), 9 (n = 26), and 12 (n = 25) months compared to healthy controls (HC, n = 20). The filled circles represent the subjects that were used for the longitudinal analysis. The cut-off value was calculated from the mean plus 2 SDs of the OD for HC. AC, acute phase; HC, healthy controls

The cross-sectional survey of MSP8 antigenicity was conducted during the acute and recovery phases of infection (Table 1). During symptomatic malaria, anti-PvMSP8 IgG levels were significantly higher than levels in malaria-naïve persons (Fig. 1). During follow-up, positive antibody responses to PvMSP8 in previously *P. vivax*-infected patients (3, 9, and 12 months after infection) were found in high numbers of subjects. Approximately 89%, 62% and 84% of these subjects were seropositive at 3, 9, and 12 months, respectively (Fig. 1). Based on these data, patients tended to maintain serological responses to PvMSP8 after recovery from infection. To add to this, the antibody responses in *P. vivax*-infected individuals were analysed over a longer post-infection period.

### Prevalence of anti-IgG PvMSP8 antibodies through 4 years post-infection

**Fig. 2:**
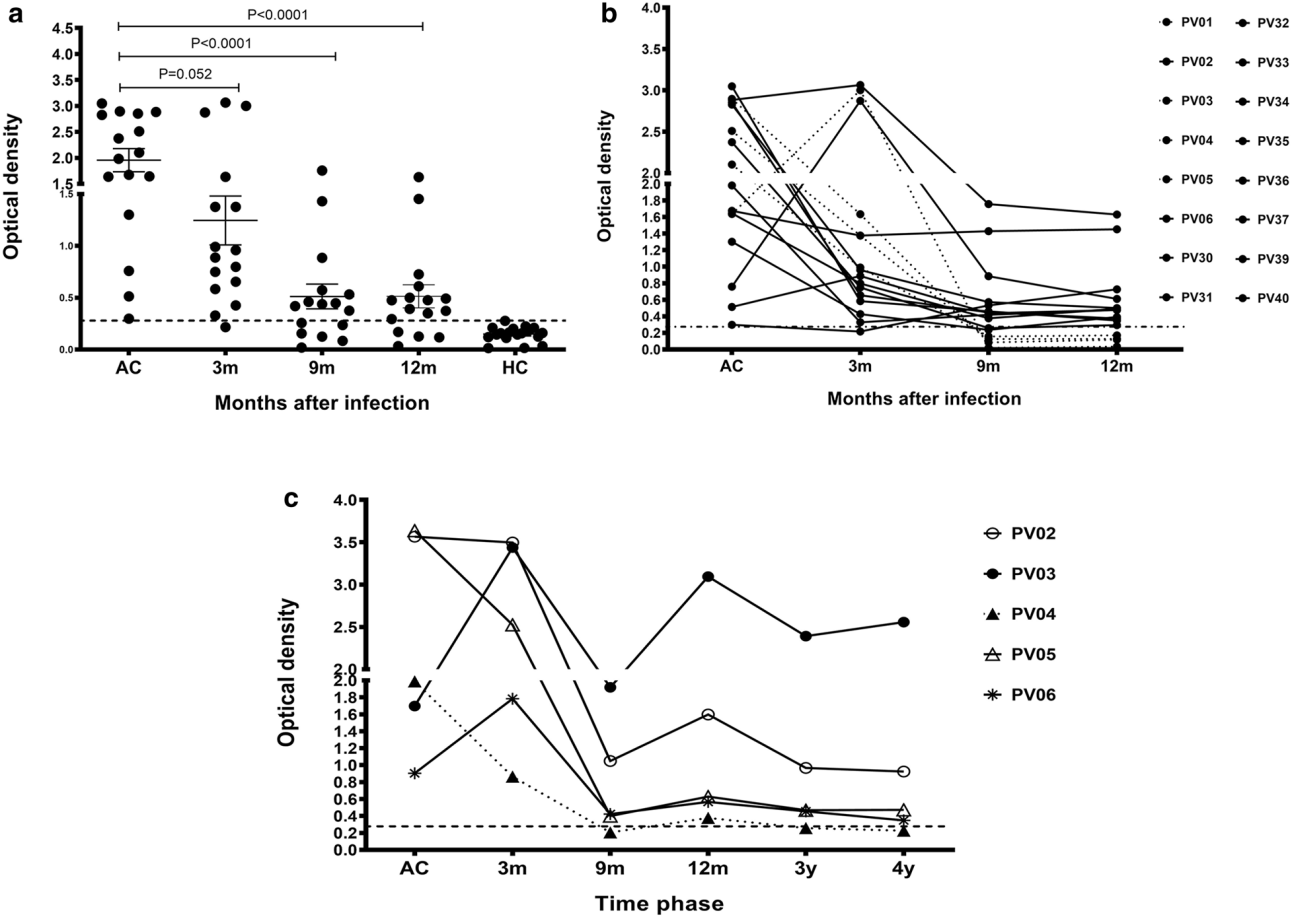
Longitudinal analysis of anti-PvMSP8 responses. The longevity of anti-PvMSP8 responses is shown in **a**, **b** for *P. vivax*-infected patients in the 12-month cohort study (n = 16) and **c** for subjects in the 4-year cohort study (n = 5). The cut-off value was calculated from the mean plus 2 SDs of the OD for HC

Sixteen patients in the sub-cohort 1 study were analysed to detect anti-PvMSP8 antibody levels and determine the longevity of anti-PvMSP8 responses after infection (Table 2). Although anti-PvMSP8 IgG levels were significantly reduced at 9 months post-infection compared to the acute phase, 63% of samples remained seropositive (Fig. 2a). Monitoring of anti-PvMSP8 IgG levels in these individuals was continued for 12 months. Eleven patients (69%) continued to be positive for anti-PvMSP8 antibody (Fig. 2b). The persistence of long-lived anti-PvMSP8 antibody responses was further examined by following five *P. vivax*-infected patients until 4 years post-infection in the sub-cohort 2 study. These 4 patients maintained seropositivity at the 4 years time point (Fig. 2c). Based on the results of these longitudinal analyses, PvMSP8 induces antibodies, which persist after recovery from infection.

### Long-term antibody responses to specific PvMSP8 B cell epitopes following infection

**Fig. 3:**
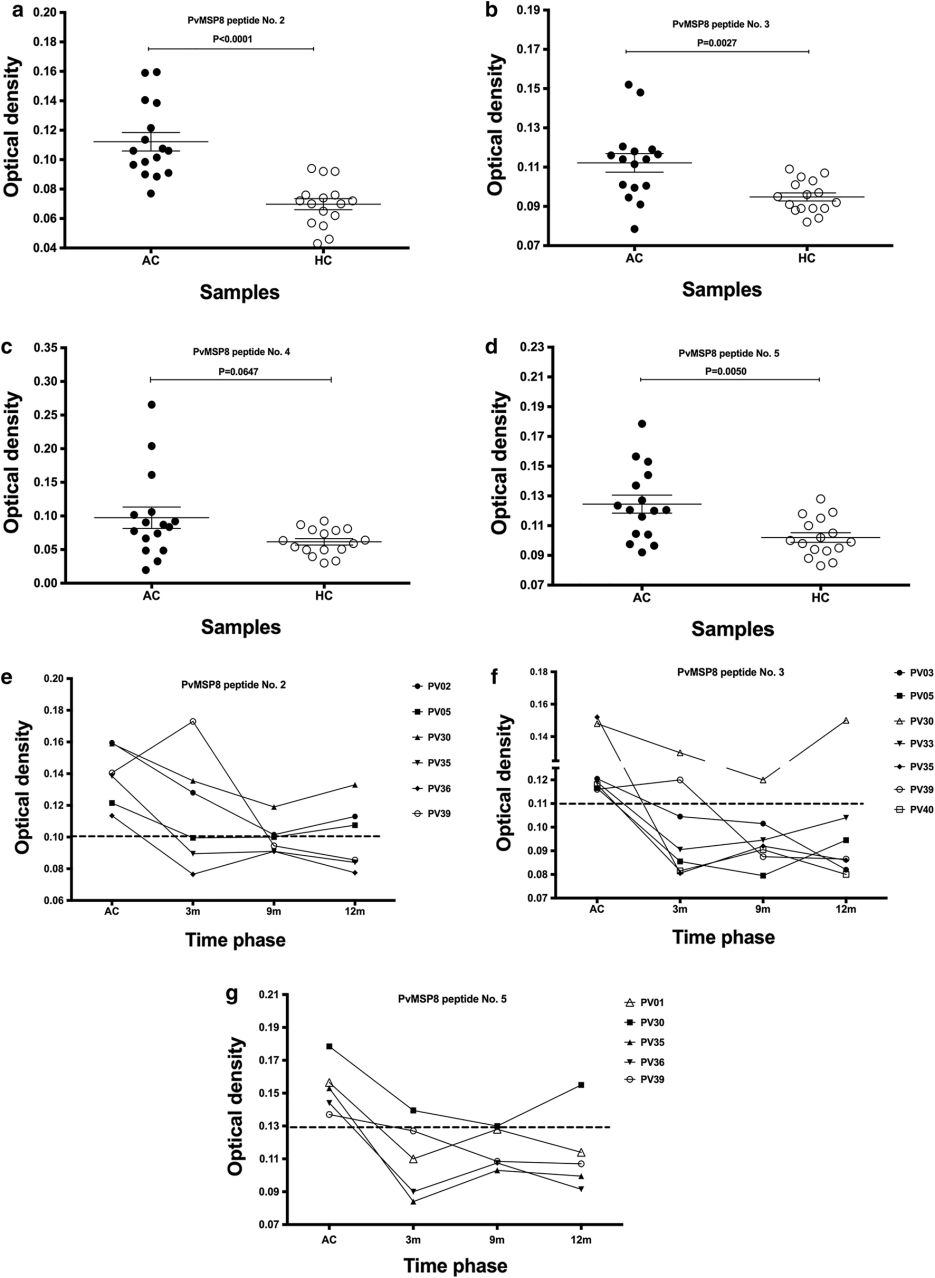
The durability of antibody responses to predominant PvMSP8 peptides. **a**–**d** The frequencies of antibody responses to PvMSP8 peptide Nos. 2, 3, 4 and 5 in patients with an acute *P. vivax* infection (n = 16) compared to HC (n = 16). **e**–**g** Antibody responses to PvMSP8 peptides in seropositive patients, peptide No. 2 (n = 6), peptide No. 3 (n = 7), and peptide No. 5 (n = 5), at acute infection and three follow-up times in the cohort study (AC, 3 months, 9 months and 12 months post-infection). The cut-off value was calculated as the mean plus 2 SDs of the OD for HC

Four predominant epitopes of PvMSP8 have been identified as targets of acquired anti-PvMSP8 antibodies in patients with an acute *P. vivax* infection [10]. It was hypothesized that the antibody responses to these B cell epitopes may persist in the long-term. In fact, the patients with acute vivax malaria produced significant antibody responses to peptide Nos. 2, 3 and 5 of the PvMSP8 protein (Fig. 3a–d) and 6 patients showed preferential recognition to one of these three peptides. Moreover, broad reactivity of anti-peptide antibodies was found as there were 2, 3 and 1 patients who were seropositive to 2, 3 and 4 peptides of PvMSP8 protein, respectively (Table 3). The further monitoring anti-PvMSP8 peptide antibody levels in seropositive patients for 12 months post-infection showed that 3 patients maintained antibody responses to peptide No. 2. Interestingly, one patient (Pv30) was found for anti-peptide Nos. 3 and 5 antibodies (Fig. 3e–g).

**Table 3:** The pattern analysis of antibody responses to synthetic MSP8 peptides in 16 patients with acute *P. vivax* infection

Subjects	Peptide No. 2	Peptide No. 3	Peptide No. 4	Peptide No. 5
PV1	−	−	−	+
PV2	+	−	−	−
PV3	−	+	−	−
PV4	−	−	−	−
PV5	+	+	−	−
PV6	−	−	−	−
PV30	+	+	+	+
PV31	−	−	+	−
PV32	−	−	−	−
PV33	−	+	+	−
PV34	−	−	−	−
PV35	+	+	−	+
PV36	+	−	+	+
PV37	−	−	+	−
PV39	+	+	−	+
PV40	−	+	−	−
Total	6	7	5	5

+ indicates positive response to one or more synthetic MSP8 peptides

− indicates negative response to synthetic MSP8 peptides

### Long-lived PvMSP8-specific MBCs were detected at 4 years post-infection

**Fig. 4:**
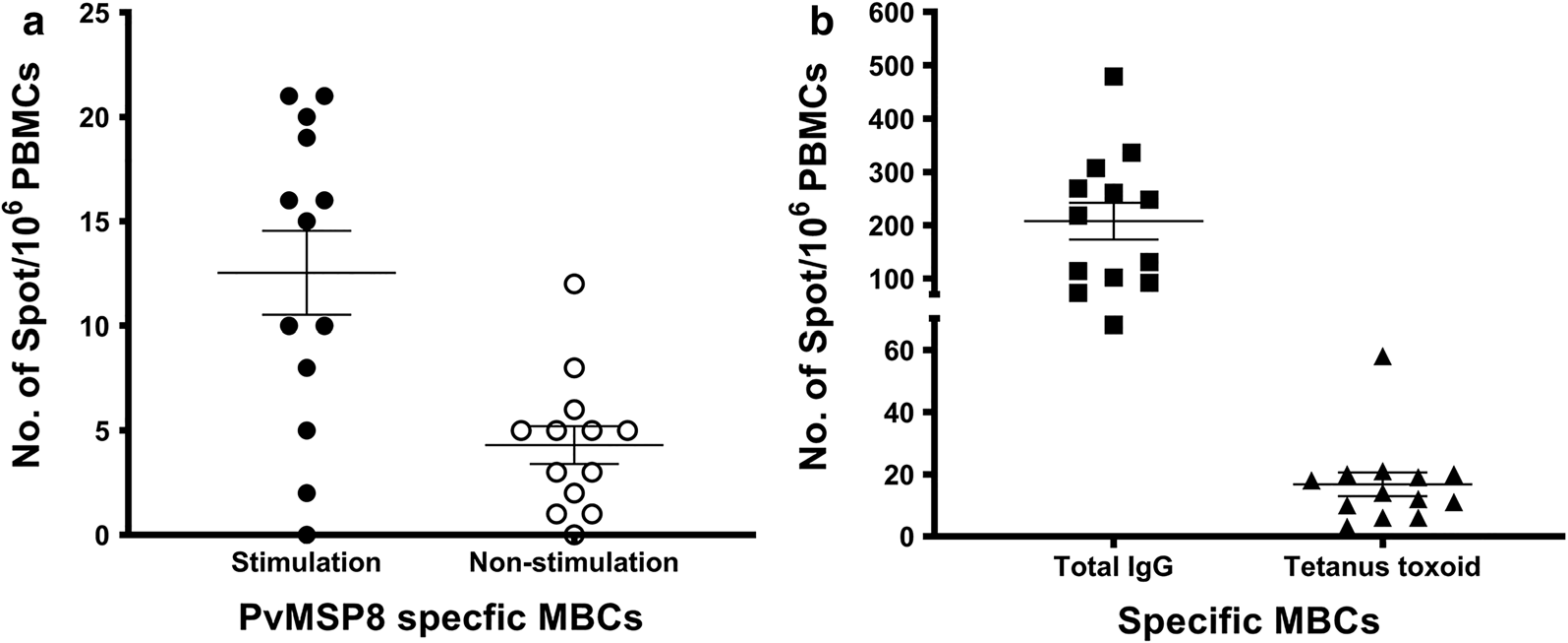
PvMSP8-specific MBC responses at 4 years post-infection. The numbers of specific MBCs produced in response to **a** PvMSP8, **b** tetanus toxoid (TT), and total IgG in PBMCs from subjects that persisted 4 years after the *P. vivax* infection were determined using an ELISPOT assay (n = 13). The frequencies of MBCs were determined by counting the number of spots that appeared per million cultured PBMCs. Each symbol represents the MBC number for one individual. The line represents the median value

The maintenance of circulating antibodies is independently related to the response of long-lived plasma cells in subjects with malaria [12]. Thus, the long-term anti-PvMSP8 antibody responses in some patients who had recovered from infection 4 years earlier were linked to the persistence of PvMSP8-specific MBCs (Table 4). Of the 13 total patients, samples from 9 patients showed positive PvMSP8-specific MBCs in the ELISPOT assay. The average frequency of PvMSP8-specific MBCs among responders was 13 PvMSP8-specific MBCs per million PBMCs, with a range of 5–21 PvMSP8-specific MBCs per million PBMCs (Fig. 4). Interestingly, PvMSP8-specific MBCs were produced in the majority (n = 9) of patients who were initially infected with *P. vivax*. Six responders who had positive PvMSP8-specific MBCs also had positive antibody titers to PvMSP8. However, the presence of long-lived MBCs was not consistently related to antibody response, as 3 seronegative subjects had positive MBC responses.

**Table 4:** The history of *P. vivax* infections in subjects enrolled in the PvMSP8-specific MBC study between 2014 and 2018

ELISPOT results	Age (mean ± SD)	Gender		No. of previous infections			No. of recorded re-infections	
		Male	Female	0	1	> 1	Symptomatic infection	Asymptomatic infection
Positive	47 ± 13.3	5	4	8	1	0	1	0
Negative	35.5 ± 15.2	1	3	4	0	0	0	0

## Discussion

The challenge in the development of a malaria vaccine is the requirement to induce long-term protective immunity. To date, the data provided by immuno-epidemiological studies have prompted debate regarding whether naturally acquired MBCs develop during an infection and whether those MBCs persist in the absence of re-infection. In this study, cross-sectional and cohort studies were designed to observe the development and persistence of antibodies and MBCs specific for the blood stage PvMSP8 antigen to address this hypothesis. The serological response to PvMSP8 was prominent during acute *P. vivax* infections as 89% of patients were seropositive to the antigen. After infection, the majority of patients maintained the anti-PvMSP8 response for up to 12 months. Interestingly, positive antibody responses were also detected in 80% of patients 4 years post-infection. Similarly, the PvMSP8-specific MBCs were positive in approximately 69% of *P. vivax*-infected subjects 4 years after their infections. Based on these data, long-term humoral immune responses to PvMSP8 developed and were maintained in individuals living in this low malaria endemic region. However, the relationship between the longevity of anti-PvMSP8 responses and protection from infection and symptom severity given infection requires validation in other cohorts and exploration in model systems.

The immunogenicity of the MSP8 antigen has been reported in patients with both *P. falciparum* and *P. vivax*-induced malaria. Surprisingly, immune responses to PvMSP8 were detectable 12 years after the last malaria episode in some individuals residing in an area that was no longer endemic for malaria [5, 10]. However, a cross-sectional study might be not sufficient to confirm the results of a longitudinal immunological analysis. Therefore, cohort studies are better able to add to the understanding of the establishment and maintenance of anti-PvMSP8 antibody responses. Here, 69% of patients in the cohort study were seropositive at 12 months post-infection, representing a long-term response that developed during the acute phase. In addition, some had PvMSP8-specific MBCs which persisted for up to 4 years after infection. However, the levels of anti-PvMSP8 antibodies were not correlated with parasitaemia in 40 acutely infected patients. Although PvMSP8 is not localized on the merozoite surface, this protein is essential for constructing the parasitophorous vacuole during the ring stage by being redistributed to the parasite surface just prior to or following invasion of RBCs [5, 6]. Thus, naturally acquired anti-PvMSP8 antibody responses could be involved in (i) the inhibiting of merozoite invasion into RBCs, (ii) blocking cytoadherence of infected RBCs to endothelial cells, and/or (iii) enhancing the phagocytic activity of monocytes and macrophages [15, 16]. On the other hand, MSP8 protein may act as a parasite-specific carrier protein to enhance the production of complex malaria vaccine targets such as MSP1, apical membrane antigen 1 and DBPII. It has been reported that PfMSP8-specific protein carrier can enhance the immunogenicity of neutralizing B cell epitopes in the 19-kDa C terminal domain of PfMSP1, and has potent transmission-reducing activity upon the fusion of PfMSP8 with the Pfs25 gamete antigen [8, 17]. Insight into the longevity of responses and the protective role of anti-MSP8 antibodies will be an important information for further development of blood stage malaria vaccine.

A synthetic peptide-based vaccine encompassing multiple protective epitopes that occur in difference stages of the malaria life cycle is considered a new strategic approach in vaccine development [18]. The MSP8 protein contains immunogenic epitopes of both T and B cells. The C terminus is involved in T cell responses [7], whereas outside the C terminal EGF-like domain is a region of B cell epitopes mapped by highly reactive patient sera [10]. These data suggest that MSP8 epitopes induce both T and B cell responses upon stimulation, as during natural infection. Thus, it was these predominant epitopes [10], which were assessed further in this study to observe whether the resulting antibody responses were maintained after infection. The longevity of anti-PvMSP8 peptide responses was addressed with this 12-month cohort study. Antibody responses against peptide Nos. 2, 3 and 5 fell over time and became seronegative at 9 months post-infection. Only 3 patients and 1 patient maintained detectable positive antibody responses against peptide Nos. 2 and 3, respectively. These three peptides, in the context of combinations or fusions of PvMSP8 peptides, may enhance or synergize long-term immune responses against blood stage antigens and should be considered for further study and possible vaccine design.

The maintenance of long-term humoral immunity against PvMSP8 in the absence of repeated antigen stimulation was demonstrated in the present study. Nine of 13 patients showed long-lived MSP8-specific MBCs at 4 years post-infection in the cohort study. Among the positive responders, 8 patients were experiencing their first *P. vivax* infection, an observation consistent with previous reports of Peruvian patients who produced MBCs specific to PfMSP1 [19] and Thai *P. vivax*-infected patients who produced MBCs specific to PvMSP1 paralog-19 and PvDBPII [11, 20]. Thus, individuals living in regions with low rates of malaria transmission might generate PvMSP8-specific MBCs even after a single infection. Interestingly, the boosting capacity of the humoral immune system against PvMSP8 may have been demonstrated by subject Pv03 whose *P. vivax* re-infection was associated with a high frequency of MSP8-specific MBCs (approximately 21 spots per million cells) (Fig. 3c). Moreover, MBCs specific to synthesized peptides Nos. 2 and 4 were detected in individuals who were ELISPOT positive to rPvMSP8, indicating that long-lived MBCs were acquired following natural exposure to the *P. vivax* parasite.

The goal of effective vaccine development is to produce a safe product, which induces or stimulates long-term protection. In studies of malaria, longitudinal cohort-designed studies of individuals who live in endemic regions are required to obtain a better understanding of the long-term immunity, which occurs in response to natural infections. Here, the presence of long-lived antibodies and MBCs specific to the PvMSP8 protein was examined using both cross-sectional and longitudinal studies. In the 12-month cohort study, seropositive antibody responses were maintained in a majority of *P. vivax*-infected patients, and these responses persisted for up to 4 years in some patients. In the 4-year cohort study, the antibody responses were related to the presence of PvMSP8-specific MBCs. Encouragingly, this study provided evidence that patients developed long-term humoral immune responses to the malaria antigen PvMSP8. Thus, this antigen might be added to existing, but only partially effective, candidate vaccines to develop products with greater efficacy.

The present study was limited by the inability to recruit a large number of *P. vivax* subjects into a cohort study; 16 and 13 patients were enrolled and followed for 1- and 4 years, respectively. Thus, these observations should be considered preliminary in their demonstration that anti-PvMSP8 antibodies and specific MBCs are stably presence in the absence of reinfection. A study with a larger sample size and improved surveillance would help to confirm these findings of long-term protective immunity induced by this *P. vivax* malaria antigen.

## Conclusions

This study reveals the antigenicity of the PvMSP8 protein and its ability to induce long-term humoral immune responses. These anti-PvMSP8 responses generally persisted for at least 12 months post-infection and were maintained in some patients for up to 4 years. Importantly, the persistence of the antibody response was related to the presence of PvMSP8-specific MBCs in the patient’s antibody positive at 4 years post-infection. In sum, PvMSP8 antigen-specific antibodies and MBCs developed and persisted in individuals living in an area of low malaria transmission.

## Data Availability

The datasets used and/or analysed during the current study are available from the corresponding author upon reasonable request.

## Abbreviations

MBCs: memory B cells
DBPII: Duffy binding protein region II
MSP8: merozoite surface protein-8
ELISA: enzyme-linked immunosorbent assay
ELISPOT: enzyme-linked immunospot assay
PBMCs: peripheral blood mononuclear cells
